# Identifying SSR Markers Related to Seed Fatty Acid Content in *Perilla* Crop (*Perilla frutescens* L.)

**DOI:** 10.3390/plants10071404

**Published:** 2021-07-09

**Authors:** Hyeon Park, Kyu Jin Sa, Do Yoon Hyun, Sookyeong Lee, Ju Kyong Lee

**Affiliations:** 1 Department of Applied Plant Sciences, College of Agriculture and Life Sciences, Kangwon National University, Chuncheon 24341, Korea; znfnfn325@naver.com (H.P.); sakajin49@kangwon.ac.kr (K.J.S.); 2 Interdisciplinary Program in Smart Agriculture, Kangwon National University, Chuncheon 24341, Korea; 3 National Agrobiodiversity Center, National Institute of Agricultural Sciences, RDA, Jeonju 54874, Korea; dyhyun@korea.kr (D.Y.H.); xsanta7@korea.kr (S.L.)

**Keywords:** *Perilla* crop, seed oil, five fatty acids, SSR marker, population structure, association analysis, MAS breeding programs

## Abstract

*Perilla* seed oil has been attracting attention in South Korea as a health food. Five fatty acids of 100 *Perilla* accessions were identified as follows: palmitic acid (PA) (5.10–9.13%), stearic acid (SA) (1.70–3.99%), oleic acid (OA) (11.1–21.9%), linoleic acid (LA) (10.2–23.4%), and linolenic acid (LNA) (54.3–75.4%). Additionally, the 100 *Perilla* accessions were divided into two groups (high or low) based on the total fatty acid content (TFAC). By using an association analysis of 40 simple sequence repeat (SSR) markers and the six *Perilla* seed oil traits in the 100 *Perilla* accessions, we detected four SSR markers associated with TFAC, five SSR markers associated with LNA, one SSR marker associated with LA, two SSR markers each associated with OA and PA, and four SSR markers associated with SA. Among these SSR markers, four SSR markers (KNUPF14, KNUPF62, KNUPF72, KNUPF85) were all associated with TFAC and LNA. Moreover, two SSR markers (KNUPF62, KNUPF85) were both associated with TFAC, LNA, and OA. Therefore, these SSR markers are considered to be useful molecular markers for selecting useful accessions related to fatty acid contents in *Perilla* germplasm and for improving the seed oil quality of *Perilla* crop through marker-assisted selection (MAS) breeding programs.

## 1. Introduction

*Perilla frutescens* (L.) Britt. is widely distributed and cultivated in the Himalayan hills and Eastern and Southeast Asian countries, but it is widely cultivated and mainly used in East Asia [1,2,3,4,5]. This species is self-fertilizing and an annual plant of the Lamiaceae family, and it is classified into two cultivated types (or varieties), *P. frutescens* var. *frutescens* and var. *crispa*, based on their morphological characters and use [1,6]. In East Asia, *P. frutescens* var. *frutescens* is used as both a seed oil and a leafy vegetable crop (ren in Chinese, dlggae in Korean, and egoma in Japanese), while *P. frutescens* var. *crispa* is used in herbal medicine and as a vegetable crop (zisu in Chinese, cha-jo-ki in Korean, and shiso in Japanese) [1,2,3]. Additionally, for centuries in East Asian countries, seed oil of *P. frutescens* var. *frutescens* has been used for foods such as cooking oils, such as soybean, rapeseed, corn, and sesame seed oils, and for industrial applications such as ink, paint, waterproofing agent, and varnish [7,8,9,10].

Meanwhile, the seeds of a cultivated type of *P. frutescens* var. *frutescens* are a good source of polyunsaturated fatty acids. *Perilla* seed oil of *P. frutescens* var. *frutescens* comprises approximately 40% of *Perilla* seed mass, and polyunsaturated fatty acids of *Perilla* seed oil, such as linoleic acid (18:2) and α-linolenic acid (18:3), comprise approximately 80% of *Perilla* seed oil. These polyunsaturated fatty acids show health benefits and have a slightly higher content than in flaxseed oil and chia seed oil, accounting for more than 60% of the total fatty acids of *Perilla* seed oil. In addition, *Perilla* seed oil contains a higher proportion of omega-3 fatty acids (54–64%) than other vegetable oils. Additionally, the omega-6 (linoleic acid) component is usually around 14%, and omega-9 (oleic acid) is also present in *Perilla* seed oil [11,12,13].

Many researchers have reported that the *Perilla* seed oil of cultivated *P. frutescens* var. *frutescens* contains polyunsaturated fatty acids such as ω-3 fatty acids (alpha-linolenic acid), ω-6 fatty acids (linoleic acid), and ω-9 fatty acids (oleic acid) [14,15,16,17,18]. These are most beneficial to human health in the prevention and control of various diseases such as cardiovascular disorders, cancer, inflammation, rheumatoid arthritis, and mental health conditions. Therefore, the *Perilla* seed oil of cultivated *P. frutescens* var. *frutescens* has attracted attention in South Korea as a health food. This has led to a significant expansion in the cultivation area of cultivated *P. frutescens* var. *frutescens*, making it one of the most important crops in South Korea.

To provide successful breeding programs for *Perilla* in South Korea, information about the seed oil content among *Perilla* germplasm accessions is very important for the development of *Perilla* oil varieties using the preserved *Perilla* genetic resources of the Rural Development Administration Genebank (RDA-Genebank) collection from South Korea. In particular, in order to maximize the efficient utilization of *Perilla* germplasm resources, it is necessary to accurately evaluate the *Perilla* germplasm accessions. Recently, DNA molecular marker systems, such as restriction fragment length polymorphisms (RFLPs) [19], randomly amplified polymorphic DNA (RAPD) [20], amplified fragment length polymorphism (AFLP) [21], simple sequence repeat (SSR) [22], and single nucleotide polymorphisms (SNPs) [23], have provided useful information on genetic diversity and relationships, population structure, and association mapping between and within accessions of many crop species and their wild species [24,25,26,27]. Furthermore, in order to develop molecular markers related to agricultural traits following the development of statistical analysis and molecular breeding technology, association mapping was proposed as a method to identify loci related to the inheritance of complex traits in many crop species [28,29]. Among the many molecular marker systems, in particular, the SSR marker system has provided useful information for the analysis of genetic diversity and relationships, population structure, genetic mapping (construct linkage maps), and association mapping in many crop species because of high reproducibility, allelic variation, codominant nature, and abundant crop genomes [22,24,27,30,31]. Recently, in the *Perilla* crop, SSR primer sets have been developed by many researchers [32,33,34,35,36]. They have successfully analyzed genetic diversity and relationships, population structure, and association mapping among the accessions of cultivated and weedy types of *Perilla* crop [4,5,26,34,36,37,38,39].

Therefore, in this study, we estimated the content of five fatty acids of *Perilla* accessions and used *Perilla* SSR primers to determine the genetic variation and population structure of *Perilla* accessions that showed high and low values in fatty acid composition. Additionally, we studied association mapping to identify molecular markers related to the content of the five fatty acids in the *Perilla* accessions, which would allow better accession selection in molecular breeding programs for the development of useful seed oil varieties of *Perilla* crop using marker-assisted selection (MAS).

## 2. Results

### 2.1. Fatty Acid Composition in Perilla Accessions

Table 1 showed the differences in the content of five fatty acids among 100 *Perilla* accessions collected from South Korea. As a result, the content of the five fatty acids in the 100 *Perilla* accessions was found to be 5.10–9.13% for PA, 1.70–3.99% for SA, 11.1–21.9% for OA, 10.2–23.4% for LA, and 54.3–75.4% for LNA. In addition, for the two groups of *Perilla* accessions, the content of the five fatty acids was found to be 6.52–9.13% for PA, 2.56–3.99% for SA, 15.6–21.9% for OA, 10.2–23.4% for LA, and 65.9–75.4% for LNA in the 50 *Perilla* accessions in Group I (Supplementary Table S1a); and 5.10–7.85% for PA, 1.70–3.53% for SA, 11.1–18.8% for OA, 2.5–22.7% for LA, and 54.3–62.3% for LNA in the 50 *Perilla* accessions in Group II (Supplementary Table S1b). Among the 100 *Perilla* accessions used for the analysis, in particular, the IT105801, IT105936, and IT157587 accessions showed the highest TFAC (120%) compared with the other accessions, while the IT117093, IT117133, and IT157549 accessions showed the lowest TFAC (97.3% or less) compared with the other accessions (Table 1).

In addition, for the five fatty acids measured in the 100 *Perilla* accessions, the content values for PA were in the range of 5.10–9.13%, with an average value of 7.43% and the highest value in the IT157587 accession and the lowest in the IT177137 accession. For SA, the values were in the range of 1.70–3.99%, with an average value of 3.15% and the highest value in the IT157489 accession and the lowest in the IT209222 accession. For OA, the values were in the range of 11.1–21.9%, with an average value of 16.3% and the highest value in the IT117033 accession and the lowest in the IT177137 accession. For LA, the values were in the range of 10.2–23.4%, with an average value of 16.2% and the highest value in the IT157587 accession and the lowest in the IT117015 accession. For LNA, values were in the range of 54.3–75.4%, with an average value of 64.6% and the highest value in the IT117153 accession and the lowest in the IT117015 accession. Furthermore, for TFAC, the values were in the range of 97.1–124.8%, with an average value of 107.6% and the highest value in the IT157587 accession and the lowest in the IT117133 accession (Table 1).

Correlation analysis was performed to evaluate the relationships among the five fatty acid contents and total fatty acid content in the 100 accessions of cultivated *P. frutescens* var. *frutescens*. Among all combinations, the combinations of PA and SA (0.315 **), PA and LA (0.246 *), PA and TFAC (0.303 **), SA and OA (0.619 **), SA and LNA (0.477 **), SA and TFAC (0.503 **), OA and LNA (0.724 **), OA and TFAC (0.829 **), LA and TFAC (0.417 **), and LNA and TFAC (0.929 **) showed a higher positive correlation coefficient than the other combinations, with a significance level of 0.01 or 0.05. The SA and LA combination (−0.157) showed the only negative correlation compared with the other combinations (Table 2).

### 2.2. Genetic Variation in the Perilla Accessions Using SSR Markers

The genetic variation for 40 SSR loci was measured with regard to the number of alleles, genetic diversity (GD), polymorphic information content (PIC), and major allele frequency (MAF) among the 100 accessions of cultivated *P. frutescens* var. *frutescens* (Table 3). The 40 SSR loci were confirmed to have a total of 231 alleles in the 100 accessions of cultivated *P. frutescens* var. *frutescens*. The number of alleles per locus ranged from 3 (KNUPF26, KNUPF58, KNUPF74, KNUPF77) to 12 (KNUPF89), and the average number of alleles per locus was 5.8 (Table 4). The average GD was 0.614, with a range of 0.313 (KNUPF11)–0.847 (KNUPF113). The average PIC value was 0.570, with a range of 0.293 (KNUPF11)–0.830 (KNUPF113). The average MAF was 0.522, with a range of 0.270 (KNUPF117, KNUPF125)–0.820 (KNUPF4) (Table 3).

### 2.3. Population Structure and Association Analysis among 100 Perilla Accessions Using SSR Markers and Fatty Acid Content

**Figure 1:**
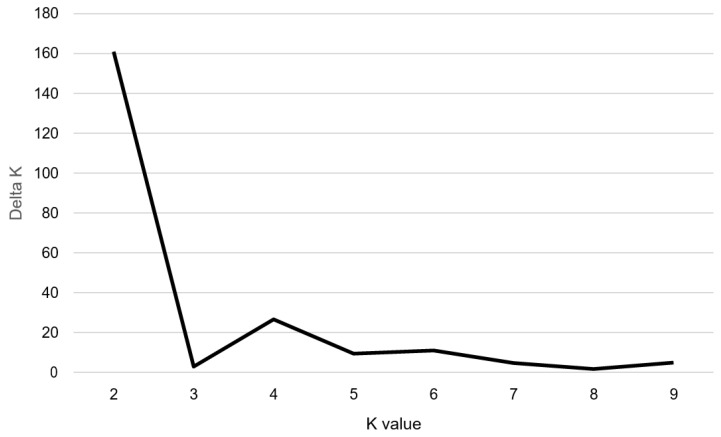
The magnitude of ∆*K* as a function of *K*; the peak value of ∆*K* was at *K* = 2, suggesting two genetic clusters in the 100 *Perilla* accessions.

**Figure 2:**
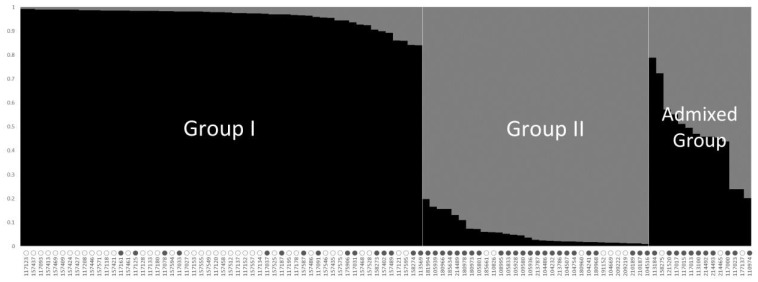
The population structure pattern for the highest Δ*K* value, *K* = 2, of 100 accessions of the cultivated var. *frutescens* based on 40 SSR markers. ○: accessions of cultivated var. *frutescens* with a low total fatty acid content, ●: accessions of cultivated var. *frutescens* with high total fatty acid content.

To understand the population structure among the 100 accessions of cultivated *P. frutescens* var. *frutescens*, we divided each accession into corresponding subgroups using the model-based approach in STRUCTURE software. The highest value of Δ*K* for the 100 accessions of cultivated *P. frutescens* var. *frutescens* was for *K* = 2 (Figure 1). As a result, all accessions were divided into two main groups and an admixed group at *K* = 2 (Figure 2). At *K* = 2, Group I included 55 accessions of the cultivated *P. frutescens* var. *frutescens*, of which 15 accessions had high total fatty acid content, and 40 accessions had low total fatty acid content. Group II included 31 accessions of the cultivated *P. frutescens* var. *frutescens*, of which 24 accessions had high total fatty acid content, and seven accessions had low total fatty acid content. The admixed group contained a total of 14 accessions of the cultivated *P. frutescens* var. *frutescens*, 11 of which had a high total fatty acid content, and three accessions had a low total fatty acid content (Figure 2). Most of the accessions with high fatty acid content were included in Group II and the admixed group.

In addition, to select the SSR markers associated with the five fatty acids in the 100 accessions of cultivated *P. frutescens* var. *frutescens*, the genotypes of the 40 SSR markers and the *Perilla* seed oil traits, namely PA, SA, OA, LA, LNA, and TFAC, were used to confirm significant marker-trait associations (SMTAs) using TASSEL software. From the results, we detected 11 SSR markers (KNUPF10, KNUPF14, KNUPF16, KNUPF53, KNUPF62, KNUPF71, KNUPF72, KNUPF85, KNUPF89, KNUPF118, and KNUPF125) associated with the six *Perilla* seed oil traits using a GLM at a significance level of *p* ≤ 0.01 or 0.001 (Table 4). Among the 11 SSR markers related to the *Perilla* seed oil traits, four SSR markers (KNUPF14, KNUPF62, KNUPF72, and KNUPF85) were associated with the TFAC trait at a significance level of *p* ≤ 0.01 or 0.001. Five SSR markers (KNUPF14, KNUPF53, KNUPF62, KNUPF72, and KNUPF85) were associated with the LNA trait at a significance level of *p* ≤ 0.01 or 0.001 (Table 4). Only one SSR marker (KNUPF125) was associated with the LA trait, with a significance level of *p* ≤ 0.01. Each of the OA and PA traits was associated with two SSR markers, KNUPF62 and KNUPF85, and KNUPF10 and KNUPF89, respectively, with a significance level of *p* ≤ 0.01 or 0.001. Finally, four SSR markers (KNUPF10, KNUPF16, KNUPF71, and KNUPF118) were associated with the SA trait at a significance level of *p* ≤ 0.01 or 0.001 (Table 4). Among these significant markers related to *Perilla* seed oil traits, the SSR markers KNUPF14, KNUPF62, KNUPF72, and KNUPF85 were associated with both the TFAC and LNA traits. In addition, one SSR marker (KNUPF62) was associated with each of the TFAC, LNA, and OA traits. Additionally, one SSR marker (KNUPF10) was associated with both the PA and SA traits (Table 4).

### 2.4. Genetic Verification of SSR Markers among 100 Perilla Accessions

**Figure 3:**
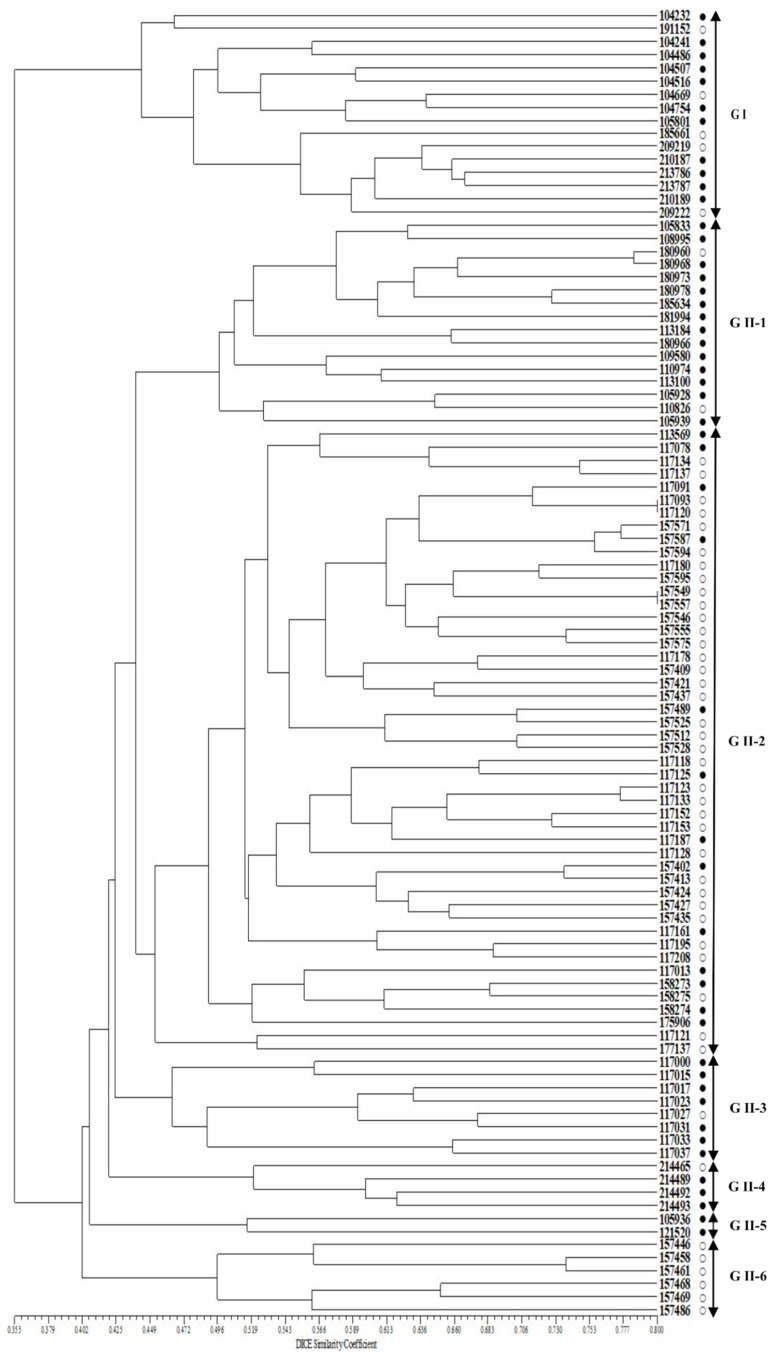
UPGMA dendrogram of 100 accessions of cultivated var. *frutescens* based on 40 SSR markers. ○: accessions of cultivated var. *frutescens* with a low total fatty acid content, ●: accessions of cultivated var. *frutescens* with high total fatty acid content.

A phylogenetic tree constructed using UPGMA and 40 SSR markers revealed the 100 *Perilla* accessions clustering into two major groups with a genetic similarity of 40.2% (Figure 3). Group I contained 16 accessions of the cultivated var. *frutescens.* Group II contained 84 accessions of the cultivated *P. frutescens* var. *frutescens.* In addition, Group II was divided into six subclusters with a genetic similarity of 44.9% (Figure 3). The first subcluster (G II-1) contained 16 accessions. The second subcluster (G II-2) contained 48 accessions. The third subcluster (G II-3) contained eight accessions. The fourth subcluster (G II-4) contained four accessions. The fifth subcluster (G II-5) contained two accessions. The sixth subcluster (G II-6) contained six accessions (Figure 3). According to our results, the clustering patterns could not clearly distinguish the accessions of cultivated *P. frutescens* var. *frutescens* to match fatty acid content, such as high or low fatty acid content. However, the groups G I, G II-1, G II-3, and G II-4 contained comparatively many accessions with high fatty acid content.

**Figure 4:**
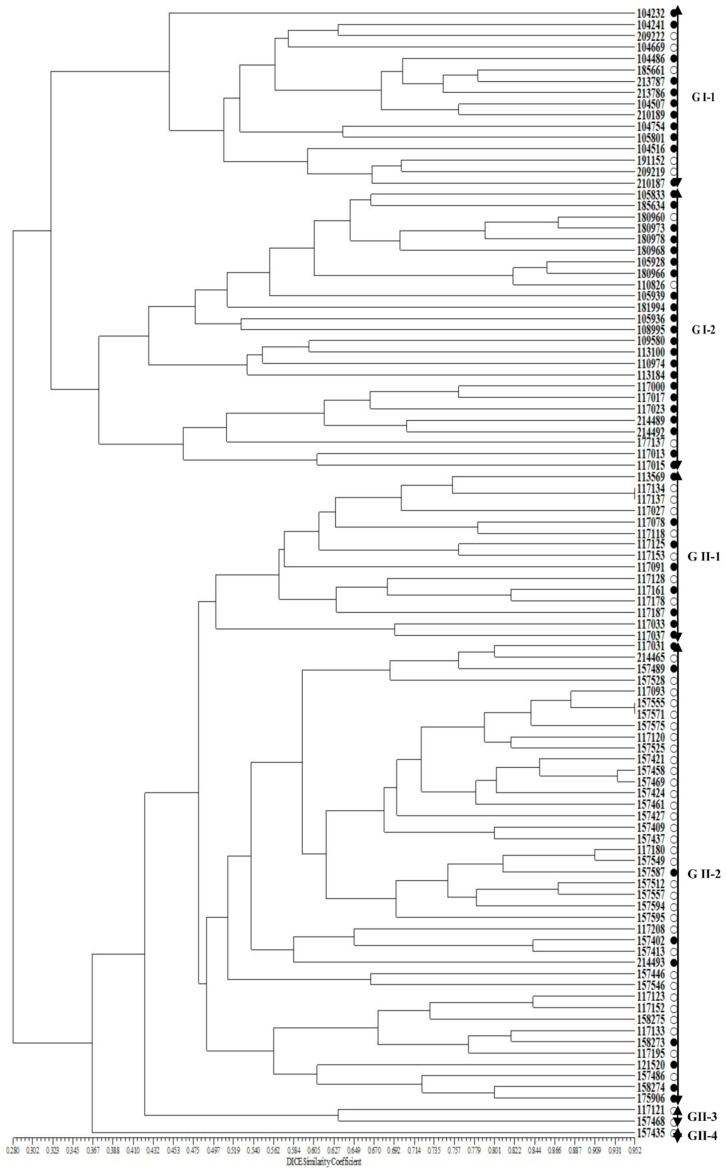
UPGMA dendrogram of 100 accessions of cultivated var. *frutescens* based on 11 SSR markers related to *Perilla* seed oil traits. ○: accessions of cultivated var. *frutescens* with a low total fatty acid content, ●: accessions of cultivated var. *frutescens* with a high total fatty acid content.

In addition, to verify the selected SSR markers associated with the *Perilla* seed oil traits among the 100 *Perilla* accessions, a UPGMA dendrogram was re-constructed using the 11 SSR markers (Figure 4). The phylogenetic tree constructed using UPGMA revealed the 100 *Perilla* accessions clustering into two major groups with a genetic similarity of 29.3%. Group I included 41 accessions of the cultivated *P. frutescens* var. *frutescens* consisting of 33 accessions with high fatty acid content and eight accessions with low fatty acid content. Group II contained 59 accessions of the cultivated *P. frutescens* var. *frutescens.* In addition, Group I was divided into two subclusters with a genetic similarity of 36.7% (Figure 4). The first subcluster (G I-1) contained 16 accessions consisting of 11 accessions with high fatty acid content and five accessions with low fatty acid content. The second subcluster (G I-2) contained 25 accessions consisting of 22 accessions with high fatty acid content and three accessions with low fatty acid content. Group II was divided into four subclusters with a genetic similarity of 44.9% (Figure 4). The first subcluster (G II-1) contained 15 accessions consisting of eight accessions with high fatty acid content and seven accessions with low fatty acid content. The second subcluster (G II-2) contained 41 accessions consisting of nine accessions with high fatty acid content and 32 accessions with low fatty acid content. The third subcluster (G II-3) contained only two accessions, which had low fatty acid content. The fourth subcluster (G II-4) contained only one accession, which had high fatty acid content.

## 3. Discussion

*Perilla* crop in East Asia is thought to have originated from China [1,7,40], but the two cultivated types of *Perilla* crop are widely cultivated and used in South Korea and Japan [2,3,4]. The cultivated type of *P. frutescens* var. *frutescens* is extensively cultivated in South Korea as both an oil crop and a vegetable crop [4,25,26], while the cultivated type of *P. frutescens* var. *crispa* is widely cultivated and used in Japan as a spicy vegetable crop [1,2,3,7]. Meanwhile, the seeds of the cultivated type of *P. frutescens* var. *frutescens* are used for plant oil production. They are a good source of polyunsaturated fatty acids, such as linoleic acid and α-linolenic acid, as these account for about 80% of *Perilla* seed oil. In particular, two essential fatty acids, oleic and linolenic acid, are significant fatty acids in terms of quality and quantity of *Perilla* seed oils [11,12,13,41]. In addition, *Perilla* leaves are rich in vitamins B and C and are preferred as salad vegetables and pickles in South Korea. Especially due to the increase in meat consumption and the development of various dishes, foods, and methods using fresh leaves and seed oil, leaves and seed oil of the cultivated type of *P. frutescens* var. *frutescens* are attracting attention as health foods in South Korea [4,26,38,39]. Therefore, in order to develop high-quality *Perilla* seed oil varieties, it is necessary to analyze the characteristics related to seed fatty acid content of genetic resources of *P. frutescens* var. *frutescens.*

In our study, to select SSR markers associated with *Perilla* seed oil traits in accessions of cultivated *P. frutescens* var. *frutescens*, we studied the genotypes of 40 SSR markers and the content of five fatty acids and total fatty acid content (PA, SA, OA, LA, LNA, and TFAC) in 100 *Perilla* accessions. In the *Perilla* crop, SSR markers were developed by Kwon et al. 2005, Park et al. 2008, Sa et al. 2018, 2019, and Kim et al. 2021 [32,33,34,35,36]. From among them, as a preliminary test, we selected 40 SSR primer sets that showed high allele band amplification and a clear banding pattern in *Perilla* accessions. Furthermore, the 100 *Perilla* accessions used in this study were selected based on the results of fatty acid content analysis in genetic resources for the development of leaf vegetable and seed oil cultivars and stored in the Rural Development Administration Genebank (RDA-Genebank) collection from South Korea (http://genebank.rda.go.kr/, accessed on 1 November 2020).

As described in Material and Methods, for analysis of association mapping to identify SSR markers associated with *Perilla* seed oil traits of the *Perilla* accessions, two groups of *Perilla* accessions were selected based on the total contents of five fatty acids of the 100 accessions of cultivated *P. frutescens* var. *frutescens* of *Perilla* crop. Group I (50 accessions) showed a high total fatty acid content of 115% or more, and Group II (50 accessions) had a low total fatty acid content of 100% or less (Supplementary Table S1). According to our results, among the 100 accessions of cultivated *P. frutescens* var. *frutescens*, the IT105801, IT105936, and IT157587 accessions showed the highest total fatty acid content compared with the other accessions, with a content of 120% or more. Meanwhile, the IT117093, IT117133, and IT157549 accessions showed the lowest total fatty acid content compared with the other accessions with a content of 97.3% or less (Table 1). Most of the five fatty acid composition values investigated in this study were similar to those reported in previous studies on *Perilla* seeds [8,9,12,41,42,43,44]. However, the contents of the five fatty acids measured in each *Perilla* accession tended to be high or low, depending on the *Perilla* accession. For example, the IT105801 accession, which has the highest total content of 121.5% of the five fatty acids, had relatively high PA, LA, and LNA contents compared with the other accessions, while the SC content was not high compared with that of the other accessions. As such, the contents of the five fatty acids measured in each *Perilla* accession varied depending on the *Perilla* accession (Table 1). Meanwhile, from the results of correlation analysis among the contents of the five fatty acids in the 100 accessions of cultivated *P. frutescens* var. *frutescens*, the combinations of PA and SA (0.315 **), PA and TFAC (0.303 **), SA and OA (0.619 **), SA and LNA (0.477 **), SA and TFAC (0.503 **), OA and LNA (0.724 **), OA and TFAC (0.829 **), LA and TFAC (0.417 **), and LNA and TFAC (0.929 **) showed a higher positive correlation, with a significance level of 0.01 (Table 2). In particular, OA and TFAC (0.829 **) and LNA and TFAC (0.929 **) showed high positive correlation coefficients compared with the other combinations. The SA and LA combination (−0.157) showed the only negative correlation compared with the other combinations (Table 2). Therefore, these results are expected to provide useful information for breeding studies to increase the content of the five fatty acids in the *Perilla* crop in the future.

Genetic diversity between individuals in a population or between populations in a crop species, derived from genes or environmental effects or from both, can be easily assessed using a variety of molecular markers [22,28,29]. As already explained in the Introduction, SSR markers have many advantages over other marker systems. The advantage of SSR markers is that they are highly reproducible and produce very high allelic variations even between very closely related varieties [22,24,27,38]. Therefore, in our study, we used SSR markers to determine the genetic diversity and relationships, population structure, and association mapping analysis in 100 accessions of cultivated *P. frutescens* var. *frutescens*, which were selected based on the content of five fatty acids in accessions of *Perilla* germplasm.

In the study of genetic diversity among these accessions, 40 SSR markers showed useful molecular markers for the study of genetic diversity and relationships in the 100 accessions of cultivated *P. frutescens* var. *frutescens*. A total of 231 alleles with 40 SSRs were detected segregating in the 100 accessions of cultivated *P. frutescens* var. *frutescens*, which yielded an average of 5.8 alleles per locus (Table 3). The number of alleles detected in our study should provide useful information for detecting SSR markers associated with the *Perilla* seed oil traits of PA, SA, OA, LA, LNA, and TFAC in the 100 accessions of cultivated *P. frutescens* var. *frutescens*. Previously, Sa et al. 2018, Kim et al. 2021, Ha et al. 2021, and Lim et al. 2021 reported the usefulness of SSR markers in determining unique genotypes of individual accessions of *Perilla* crop and related weedy types in studies of association mapping and bulk segregant analyses [34,36,38,39]. Therefore, in our study, to select SSR markers associated with *Perilla* seed oil traits in accessions of cultivated *P. frutescens* var. *frutescens*, we analyzed marker-trait associations (SMTAs) between 40 SSR markers and six *Perilla* seed oil traits, namely PA, SA, OA, LA, LNA, and TFAC, in the 100 *Perilla* accessions using TASSEL software. From the results, we found 11 SSR markers related to *Perilla* seed oil traits at a significance level of *p* ≤ 0.01 or 0.001 as follows: four markers were associated with the TFAC trait, five markers were associated with the LNA trait, one marker was associated with the LA trait, two SSR markers were associated with both the OA and PA traits, and four SSR markers were associated with the SA trait (Table 4). Among these SSR markers related to *Perilla* seed oil traits, particularly KNUPF14, KNUPF62, KNUPF72, and KNUPF85 were all associated with the TFAC and LNA traits. In addition, one SSR marker (KNUPF62) was associated with each of the TFAC, LNA, and OA traits. Additionally, one SSR marker (KNUPF10) was associated with both the PA and SA traits (Table 4). Therefore, these SSR markers are thought to be useful molecular markers for distinguishing *Perilla* seed oil traits in the *Perilla* crop.

Furthermore, in order to understand the genetic variability and genetic relationships among accessions of the cultivated *P. frutescens* var. *frutescens* used in our study in more detail, we analyzed phylogenetic relationships for the 100 *Perilla* accessions using the 40 SSR markers and also the selected 11 SSR markers related to *Perilla* seed oil traits. In the case of the 40 SSR markers, the 100 *Perilla* accessions were clustered into two major groups with 40.2% genetic similarity (Figure 3). Group I contained 16 *Perilla* accessions consisting of 11 accessions with high fatty acid content and 5 accessions with low fatty acid content. Group II contained 84 accessions consisting of 39 accessions with high fatty acid content and 45 accessions with low fatty acid content (Figure 3). The results show that these 40 SSR markers were not clearly identified among the accessions of cultivated *P. frutescens* var. *frutescens* by high or low fatty acid content. In the case of the 11 SSR markers related to *Perilla* seed oil traits, the 100 *Perilla* accessions were clustered into two major groups with a genetic similarity of 29.3% (Figure 4). Group I included 41 accessions of the cultivated var. *frutescens* consisting of 33 accessions with high fatty acid content and eight accessions with low fatty acid content. Group II contained 59 accessions of the cultivated var. *frutescens* consisting of 17 accessions with high fatty acid content and 42 accessions with low fatty acid content (Figure 4). The results show that most accessions with high fatty acid content were clustered in Group I rather than Group II. According to our results, although some accessions were still mixed in the UPGMA dendrogram of Figure 4, when divided into sub-groups, the 11 SSR markers related to the *Perilla* seed oil traits showed a clear distinction between accessions with high or low fatty acid contents compared with the cluster results using the 40 SSR markers. Therefore, these SSR markers related to the *Perilla* seed oil traits are expected to be useful in selecting *Perilla* accessions for the content of the five fatty acids.

One particular finding in our study was that, of the 11 selected SSR markers related to the *Perilla* seed oil traits, four SSR markers (KNUPF14, KNUPF62, KNUPF72, KNUPF85) were all associated with the two traits of TFAC and LNA. Additionally, 2 SSR markers (KNUPF62, KNUPF85) were both associated with the three traits of TFAC, LNA, and OA (Table 4). In addition, in the correlation analysis between the five fatty acid contents, the combination of LNA and TFAC (0.929 **) showed the highest positive correlation coefficients compared with the other combinations. Furthermore, the combinations of OA and LNA (0.724 **) and OA and TFAC (0.829 **) showed high positive correlation coefficients compared with the other combinations (Table 2). Therefore, these results suggest that these SSR markers, particularly KNUPF62 and KNUPF85, are useful molecular markers for selecting accessions of cultivated *P. frutescens* var. *frutescens* with high or low content of fatty acids such as TFAC, LNA, and OA. Meanwhile, among the 11 SSR markers found in this study, three SSR markers (KNUPF10, KNUPF16, and KNUPF85) were previously reported by previous studies of Ha et al. 2021 and Lim et al. 2021 [38,39]. They reported that the SSR marker KNUPF85 was associated with seed characteristics such as germination rate, seed hardness, and seed size (Ha et al. 2021) [38]. Additionally, the two SSR markers, KNUPF10 and KNUPF16, were associated with leaf- and seed-related traits, respectively (Lim et al. 2021) [39]. Therefore, these SSR markers are thought to be useful molecular markers for selecting accessions related to fatty acid content and seed- or leaf-related traits in *Perilla* genetic resources. Meanwhile, for the 11 SSR markers associated with the *Perilla* seed oil traits selected in this study, there is still a still lack of genomic information on the *Perilla* crop, making it difficult to compare genetic characteristics.

Recently, the *Perilla* seed oil of *P. frutescens* var. *frutescens* has been attracting attention as a health food, and the cultivation area of cultivated *P. frutescens* var. *frutescens* has expanded significantly, making it one of the most important crops in South Korea. However, even though it is one of the representative minor crops in South Korea, there is a lack of genomic studies compared with other important crops. Therefore, if active research such as genome analysis is carried out in the *Perilla* crop, it is expected that the characteristics of the selected *Perilla* SSR markers can be specifically analyzed. The *Perilla* SSR markers found in our study are expected to be useful markers for genetic diversity analysis of *Perilla* germplasm and the selection of *Perilla* accessions with high seed oil composition for improving the oil yield and quality in *Perilla* breeding. Therefore, these results are very important for understanding the *Perilla* seed oil traits of *Perilla* crop. Additionally, they may provide support for effective selection and utilization of existing accessions and allow better accession selection in molecular breeding programs for the development of useful seed oil varieties of the *Perilla* crop through MAS breeding programs.

## 4. Materials and Methods

### 4.1. Plant Materials and Fatty Acid Composition

In this study, for the analysis of the fatty acid composition of *Perilla* accessions and association mapping, a total of 100 accessions of cultivated *P. frutescens* var. *frutescens* of *Perilla* crop were selected, based on the content of five fatty acids, from among the accessions of *Perilla* germplasm that were published on the website of the RDA-Genebank of the Republic of Korea (https://genebank.rda.go.kr/, accessed on 1 November 2020). The method of fatty acid analysis performed by the Korean RDA-Genebank was presented by Song et al. 2012 [41]. When performing fatty acid analysis on *Perilla* genetic resources in the RDA-Genebank, they used the fatty acid calculation method for each accession as follows; the relative fatty acid content between *Perilla* germplasms was assessed by making a calibration graph for each fatty acid (oleic acid, linoleic acid, etc.) and using Near-Infrared Reflectance Spectroscopy (NIR). Therefore, some accessions with a very high fatty acid content had a total fatty acid content exceeding 100%, and some accessions with a relatively low fatty acid content showed a value of less than 100%. In addition, there have been many previous studies that have reported analyses of the fatty acid content for the *Perilla* crop, such as Lee et al. 1986, Shin and Kim 1994, Asif 2011, Ciftci et al. 2012, and Lee et al. 2016 [8,10,11,12,13]. The results of our study on the content of five fatty acids, namely palmitic acid (PA), stearic acid (SA), oleic acid (OA), linoleic acid (LA), and linolenic acid (LNA), for the 100 *Perilla* accessions are shown in Supplementary Table S1. In addition, for analysis of association mapping to find SSR markers associated with the content of the five fatty acids of *Perilla* seeds, we divided the 100 accessions into two groups based on total fatty acid content (TFAC) as follows: Group I showed a high total fatty acid content of 115% or more (50 accessions), and Group II had a low total fatty acid content of 100% or less (50 accessions) (Supplementary Table S1). The information on the content of the five fatty acids of the 100 *Perilla* accessions used in this study is shown in Table 1 and Supplementary Table S1.

### 4.2. SSR Analysis and DNA Electrophoresis

For SSR analysis, the total DNA of 100 *Perilla* accessions was extracted from young leaf tissue of individual representative plants of each accession according to Plant DNAzol Reagent protocols (GibcoBRL Inc., Grand Island, NY, USA). The information on the 40 *Perilla* SSR primer sets used in this study is shown in Table 5. The SSR amplification method for the *Perilla* crop has been described in a previous study by Oh et al. 2020 [26]. After polymerase chain reaction (PCR) amplification using *Perilla* SSR primer sets, DNA electrophoresis analysis was performed with a QIAxcel advanced system (QIAGEN Co., Hilden, Germany) based on the protocol described in the QIAxcel DNA Handbook. The samples were run in the QIAxcel advanced electrophoresis system, and sample separation was performed over 15 min. Gel images were obtained as the results, and the quantification analysis was performed with QIAxcel software. The results were displayed as gel images and electropherograms acquired from the QIAxcel advanced system software.

### 4.3. Data Analysis

DNA fragments amplified for each SSR primer set were scored as present (1) or absent (0). Power Marker version 3.25 [45] was applied to obtain information on the number of alleles, allele frequency, major allele frequency (MAF), gene diversity (GD), and the polymorphic information content (PIC). Genetic similarity (GS) was calculated for each pair of accessions using the Dice similarity index [46]. The similarity matrix was then used to construct a dendrogram with the unweighted pair group method with the arithmetic mean (UPGMA) by the application of SAHN clustering in NTSYS-pc V2.1 [47]. Population structure was investigated for 100 *Perilla* accessions using STRUCTURE 2.2 software [48]. Five independent runs with *K* values ranging from one to ten were performed with 100,000 cycles for both burn-in and run length. The delta *K* statistic, based on the rate of change in the log probability of data between *K* values [49], was calculated with STRUCTURE HARVESTER (http://taylor0.biology.ucla.edu/structHarvester/, accessed on 1 November 2020) based on the STRUCTURE results. Association mapping was performed for marker-trait association using TASSEL 3.0 [50], which was used to evaluate marker-trait associations using a general linear model (Q GLM). The Q GLM method was performed using the population structure (Q) matrix derived from the STRUCTURE program. The number of permutation runs was set to 10,000 to obtain a marker significance value of *p* ≤ 0.01. Correlation analysis of the content of the five fatty acids of the 100 *Perilla* accessions was performed using SPSS software.

## 5. Conclusions

In order to identify simple sequence repeat (SSR) markers associated with *Perilla* seed oil traits in *Perilla* crop (*Perilla frutescens* (L.), the genetic variation, population structure, and association mapping of *Perilla* accessions were studied using five fatty acid contents of 100 *Perilla* accessions and 40 *Perilla* SSR primers. *Perilla* seed oil of cultivated type of *P. frutescens* var. *frutescens* has been attracting attention in South Korea as a health food. Five fatty acids of 100 *Perilla* accessions were identified as follows: palmitic acid (PA) (5.10–9.13%), stearic acid (SA) (1.70–3.99%), oleic acid (OA) (11.1–21.9%), linoleic acid (LA) (10.2–23.4%), and linolenic acid (LNA) (54.3–75.4%). Additionally, the 100 *Perilla* accessions were divided into two groups (high or low) based on the total fatty acid content (TFAC). In a correlation analysis among these six *Perilla* seed oil traits of the 100 *Perilla* accessions, the combinations of OA and LNA (0.724 **), OA and TFAC (0.829 **), and LNA and TFAC (0.929 **) showed a higher positive correlation coefficient than the other combinations. By using an association analysis of 40 SSR markers and the six *Perilla* seed oil traits in the 100 *Perilla* accessions, we detected four SSR markers associated with TFAC, five SSR markers associated with LNA, one SSR marker associated with LA, two SSR markers each associated with OA and PA, and four SSR markers associated with SA. Among these SSR markers, four SSR markers (KNUPF14, KNUPF62, KNUPF72, and KNUPF85) were all associated with TFAC and LNA. Additionally, two SSR markers (KNUPF62 and KNUPF85) were both associated with TFAC, LNA, and OA. The *Perilla* SSR markers found in our study are expected to be useful markers for genetic diversity analysis of *Perilla* germplasm and the selection of *Perilla* accessions with high seed oil composition for improving the oil yield and quality in *Perilla* breeding. Therefore, these results are very important for understanding the *Perilla* seed oil traits of the *Perilla* crop. Additionally, they may provide support for effective selection and utilization of existing accessions and allow better accession selection in molecular breeding programs for the development of useful seed oil varieties of *Perilla* crop through MAS breeding programs.

## Figures and Tables

**Table 1:** The difference in the content of five fatty acids among 100 *Perilla* accessions collected from South Korea.

Accession No.			Fatty Acid Content			
						Total (%)
	Palmitic Acid (%)	Stearic Acid (%)	Oleic Acid (%)	Linoleic Acid (%)	Linolenic Acid (%)	
104232	7.39	3.47	17.9	15.4	74.4	118.6
104241	7.38	3.19	19.0	15.4	71.7	116.7
104486	7.29	3.36	17.7	16.6	70.5	115.4
104507	7.28	3.13	19.6	20.9	65.9	116.8
104516	8.18	3.26	18.4	20.3	66.4	116.6
104669	7.57	3.10	14.3	14.8	57.9	97.6
104754	8.23	3.28	19.1	22.1	65.9	118.6
105801	8.91	3.33	18.5	21.0	69.7	121.5
105833	7.09	3.41	20.9	15.2	70.5	117.1
105928	8.76	3.24	18.7	18.3	69.8	118.8
105936	7.84	3.20	18.8	15.7	75.0	120.5
105939	7.41	2.56	17.1	20.2	72.2	119.4
108995	7.07	3.14	20.4	19.3	68.1	118.0
109580	7.43	3.34	18.2	17.0	72.4	118.4
110826	7.39	2.87	14.2	15.4	58.1	97.9
110974	7.79	3.30	18.5	14.2	75.1	118.9
113100	7.54	3.21	19.1	20.1	66.3	116.2
113184	8.53	3.29	17.8	21.0	68.6	119.2
113569	7.45	3.06	18.6	20.0	68.8	117.9
117000	7.02	3.77	21.2	13.5	70.8	116.3
117013	7.08	3.47	20.4	15.3	69.5	115.8
117015	7.05	3.88	19.0	10.2	75.4	115.5
117017	6.87	3.78	20.3	19.7	67.3	117.9
117023	7.28	3.70	19.6	12.6	72.9	116.0
117027	7.26	3.37	14.2	15.2	57.9	97.9
117031	7.49	3.95	21.3	12.0	71.0	115.7
117033	6.85	3.97	21.9	15.1	68.8	116.6
117037	7.37	3.79	21.3	14.5	69.1	116.0
117078	6.91	3.62	19.8	13.3	71.7	115.4
117091	7.19	3.28	18.1	20.6	70.3	119.5
117093	7.74	3.53	13.5	15.9	56.5	97.3
117118	7.18	3.06	15.3	15.8	56.9	98.2
117120	6.95	2.91	13.6	14.4	60.3	98.2
117121	7.33	2.96	11.9	14.5	61.0	97.7
117123	7.54	3.27	11.9	17.1	57.7	97.6
117125	7.53	3.71	19.2	16.7	68.0	115.2
117128	7.54	3.24	14.2	15.1	57.7	97.8
117133	7.76	3.38	11.3	15.6	59.1	97.1
117134	7.42	2.67	13.8	16.0	57.7	97.6
117137	7.67	2.95	12.5	17.7	56.6	97.5
117152	7.84	3.22	12.6	16.9	57.1	97.7
117153	7.67	3.28	14.8	17.7	54.3	97.7
117161	8.92	3.36	17.2	16.4	70.5	116.4
117178	7.85	3.33	16.5	12.6	57.6	98.0
117180	7.63	2.86	12.8	15.5	59.3	98.0
117187	7.28	3.25	18.7	13.6	72.5	115.4
117195	7.72	3.02	17.1	14.3	55.8	97.9
117208	7.59	3.03	16.8	14.2	56.1	97.7
121520	6.52	3.72	18.5	13.8	73.8	116.3
157402	7.26	2.95	17.3	20.0	67.9	115.4
157409	7.74	3.14	13.5	14.0	59.4	97.8
157413	7.47	3.21	15.6	15.2	56.3	97.8
157421	7.70	3.02	15.8	15.2	56.4	98.1
157424	7.61	3.22	14.7	17.1	55.2	97.8
157427	7.76	3.16	13.4	13.8	59.5	97.6
157435	7.78	3.18	13.1	14.4	59.2	97.6
157437	7.55	3.09	13.9	14.5	59.0	98.0
157446	7.46	3.11	13.1	16.5	57.4	97.6
157458	7.12	2.70	14.4	14.0	59.9	98.1
157461	7.12	2.67	13.8	14.3	60.3	98.3
157468	7.38	2.71	13.7	14.3	59.7	97.8
157469	6.00	1.90	12.0	21.2	58.8	99.9
157486	6.84	2.95	18.8	13.7	57.0	99.2
157489	7.10	3.99	19.2	12.6	74.4	117.3
157512	7.60	3.17	13.8	15.8	57.3	97.6
157525	7.19	3.11	14.8	14.3	58.5	97.9
157528	7.41	2.93	12.8	14.8	59.8	97.7
157546	7.06	2.64	13.1	14.4	60.4	97.6
157549	6.98	3.19	13.7	12.7	60.7	97.3
157555	7.18	2.82	14.4	13.8	59.3	97.5
157557	7.10	2.84	13.5	13.3	60.7	97.4
157571	7.09	2.71	12.6	15.1	60.6	98.1
157575	7.06	2.49	13.2	13.5	61.8	98.1
157587	9.13	3.29	17.7	23.4	71.3	124.8
157594	6.70	2.59	14.3	12.5	62.3	98.4
157595	6.90	2.55	12.4	14.8	61.5	98.2
158273	7.01	3.01	19.7	21.0	66.0	116.7
158274	8.52	3.12	15.6	20.2	68.7	116.1
158275	7.07	3.14	14.8	15.0	57.7	97.7
175906	7.52	3.06	16.8	17.7	72.5	117.6
177137	5.10	2.10	11.1	22.7	58.9	99.9
180960	6.89	2.81	16.5	14.4	57.2	97.8
180966	7.73	3.10	16.7	16.1	73.8	117.4
180968	6.79	3.69	19.2	17.5	68.6	115.8
180973	7.78	3.14	16.2	17.9	71.1	116.1
180978	8.73	3.12	16.0	19.2	70.9	117.9
181994	7.08	3.18	18.5	17.1	73.1	119.0
185634	8.03	3.24	17.7	21.8	66.3	117.1
185661	7.05	2.95	13.8	14.2	59.9	97.9
191152	7.08	3.03	14.8	14.3	58.2	97.4
209219	7.08	2.93	12.7	14.9	60.3	97.9
209222	6.20	1.70	15.3	17.0	59.7	99.9
210187	7.33	3.34	19.0	20.6	66.6	116.8
210189	7.62	3.35	16.1	14.0	74.7	115.8
213786	8.33	3.33	17.5	16.3	71.7	117.1
213787	7.57	3.01	18.5	12.7	74.8	116.6
214465	7.22	2.87	13.5	14.4	59.9	97.9
214489	7.58	3.13	17.3	18.0	71.1	117.1
214492	8.58	3.28	16.5	17.8	68.9	115.1
214493	7.14	3.67	17.9	13.7	73.3	115.7
Mean	7.43 ± 0.592	3.15 ± 0.39	16.3 ± 2.75	16.2 ± 2.76	64.6 ± 6.44	107.6 ± 9.79
Max	9.13	3.99	21.9	23.4	75.4	124.8
Min	5.10	1.70	11.1	10.2	54.3	97.1

**Table 2:** The correlation analysis for six traits related to the fatty acid content in 100 *Perilla* accessions.

	SA	OA	LA	LNA	TFAC
PA	0.315 **	0.123	0.246 *	0.192	0.303 **
SA		0.619 **	−0.157	0.477 **	0.503 **
OA			0.141	0.724 **	0.829 **
LA				0.132	0.417 **
LNA					0.929 **

** Significance at *p* < 0.01, * Significance at *p* < 0.05.

**Table 3:** Estimates of the allele number, GD, PIC, and MAF of 40 SSR primer sets among 100 *Perilla* accessions.

SSR Loci	Allele No	GD	PIC	MAF
KNUPF1	5	0.332	0.315	0.810
KNUPF2	9	0.757	0.717	0.450
KNUPF3	6	0.625	0.585	0.630
KNUPF4	5	0.709	0.655	0.820
KNUPF5	6	0.687	0.633	0.620
KNUPF10	9	0.743	0.718	0.280
KNUPF11	4	0.313	0.293	0.550
KNUPF14	5	0.594	0.544	0.700
KNUPF16	9	0.798	0.769	0.710
KNUPF19	4	0.329	0.310	0.650
KNUPF23	4	0.502	0.457	0.570
KNUPF25	6	0.637	0.575	0.280
KNUPF26	3	0.398	0.353	0.810
KNUPF28	5	0.486	0.450	0.320
KNUPF35	4	0.635	0.579	0.670
KNUPF36	4	0.665	0.618	0.490
KNUPF37	4	0.661	0.602	0.750
KNUPF39	7	0.695	0.662	0.690
KNUPF43	5	0.645	0.574	0.560
KNUPF53	11	0.807	0.783	0.520
KNUPF55	9	0.759	0.720	0.500
KNUPF58	3	0.462	0.414	0.470
KNUPF59	5	0.543	0.457	0.490
KNUPF62	6	0.612	0.570	0.370
KNUPF71	4	0.639	0.568	0.430
KNUPF72	9	0.771	0.740	0.430
KNUPF74	3	0.516	0.453	0.310
KNUPF77	3	0.663	0.589	0.320
KNUPF78	4	0.665	0.603	0.700
KNUPF80	7	0.794	0.764	0.570
KNUPF82	6	0.697	0.643	0.570
KNUPF85	4	0.565	0.516	0.460
KNUPF89	12	0.824	0.802	0.370
KNUPF107	6	0.568	0.540	0.640
KNUPF112	8	0.588	0.567	0.370
KNUPF113	10	0.847	0.830	0.440
KNUPF117	4	0.617	0.566	0.270
KNUPF118	4	0.446	0.383	0.400
KNUPF121	4	0.461	0.424	0.610
KNUPF125	5	0.516	0.463	0.270
Mean	5.8	0.614	0.570	0.522
Total	231			

GD: Gene Diversity, PIC: Polymorphism Information Content, MAF: Major Allele Frequency.

**Table 4:** Information on SMTA markers using the GLM method for 100 *Perilla* accessions.

Trait	SSR Marker	GLM
TFAC	KNUPF14	***
	KNUPF62	***
	KNUPF72	**
	KNUPF85	***
LNA	KNUPF14	***
	KNUPF53	**
	KNUPF62	***
	KNUPF72	**
	KNUPF85	***
LA	KNUPF125	**
OA	KNUPF62	**
	KNUPF85	**
PA	KNUPF10	**
	KNUPF89	***
SA	KNUPF10	***
	KNUPF16	**
	KNUPF71	***
	KNUPF118	**

** *p* ≤ 0.01, *** *p* ≤ 0.001.

**Table 5:** Characteristics of the 40 *Perilla* microsatellite loci used in the study.

SSR Loci	Forward Primer	Reverse Primer	Repeat Motif
KNUPF1	CTTGCAGCTGATCATTAAGCTA	TTTCTTGTGTGCTCTAACAACG	(AG)11
KNUPF2	GAAACCAAATTTCTTGTTCTTACA	CAAACGCAGACTCTTATCAATG	(AG)16
KNUPF3	TTCCTTGTAGTCATCTGATCCC	TGGAAATTAATTAAAGGGCTGA	(AG)16
KNUPF4	TTTCAAAAATCTTACCAACGCT	TTCGTTTTTGCATCTAATTATTCA	(AT)10
KNUPF5	TCCATCTCATCTCATTCAAACA	ATGGATCGGAAATCTAAAAACA	(AT)10
KNUPF10	GCTGATGGGACTACCCATAATA	AGGATCGGAACAATTATTAGCC	(AT)11
KNUPF11	TTGCAAGGTAAGATGATGATGA	TTGAGGATTGACAATGTTCGT	(CA)10
KNUPF14	AAATTCTCCCTCCACTCTTCAC	TGTTGGCTTTTTCAAATCTTTT	(CT)12
KNUPF16	CCTGTATCTCTCCCCGATAAAT	TGGATTTAATGCAGTTGAGTTG	(CT)22
KNUPF19	TCGAGGTTGAACAGATACAATG	TGATTAATTTCTTACGTACACTCCA	(TG)10
KNUPF23	TTGCAAGTTCTTGAATTGTGAC	CACTCCTTCCCTCCTCTTTAAT	(TG)11
KNUPF25	GCTTAGTGTGAGGAATTATGTAGGA	ACTCAGCATGCTTGAATTCTC	(AAG)12
KNUPF26	ATTTGAAATCGAAAAAGCAAAA	TGCAACCCTATTAGCAGTTTCT	(ACA)8
KNUPF28	CAACCTCTTAAGCCTTTGAACA	AATGTGACGGGTTCTGTAAATC	(AGA)8
KNUPF35	CTCTTTCCTTCTCATTCACCAC	CCCTTTTTCTTACCCACTCTCT	(CTT)10
KNUPF36	GGGAGACGAGATAACACATGAT	TGCATACTCGATTGAAAGAAGA	(GCT)8
KNUPF37	GGTGTGAAAAAGAGAGTGGAGA	TTGAATTGCCTGTTGATAGTGA	(GGT)10
KNUPF39	TCACCTTCCCCTTCATTTATTA	AGGATCGAACAGAACAAACTGT	(TCT)13
KNUPF43	GTCAAATGAAATTCACACATTTTA	GTAAATGGGAATTTTTGAGGAG	(AT)7
KNUPF53	GATTCATCATTCAGCTCTCTCC	ATGACCAATGGATTAAACAAGG	(CT)17
KNUPF55	TGCTGTTGATGACTTGTATGGT	ATGAGATTTGGCTTCACAGAGT	(AGC)7
KNUPF58	GTATATGTGTGGGAAGGTTGCT	TCAATTTCCTCATCAAATCAAA	(ATG)7
KNUPF59	AATCTCGATGCCTAACAACAGT	TTCCTTGTAAATCCAGCTAAGG	(CAG)7
KNUPF62	CCATCCTTCTTGTTCAACTCAT	AATGTTGATGAGGAGACGTTTT	(CAT)7
KNUPF71	GAAGAATGCATCAGTAACACGA	ATGCTGGCCAAGTAATAAGAGA	(AGCT)4
KNUPF72	TAATTTGAGGGATTCCTTTCCT	CGCCACCCTTACTACTTCATAC	(TCGA)4
KNUPF74	TTGACTGTACCAGAGCATCAAG	GGGTACACTCACAACTCTACCAA	(AAAT)6
KNUPF77	TTTTTGGTTGCTTTTTCTTGAT	AGCAGATAAAATGTGCTGGATT	(TATG)10
KNUPF78	GCGTTATTATTTTTCAAGATCG	TCAATGATTTTACAGAAGATGCT	(AAC)7
KNUPF80	GATTCATCATTCAGCTCTCTCC	ATGACCAATGGATTAAACAAGG	(CT)17
KNUPF82	AAACCAAGGAACTCGTCAACTA	CGCTTCGTCTTTATTGTGTGTA	(AGA)7
KNUPF85	GATGACGATGAAGCTTTTCAG	CTCCTCAGCAGTTTCACCTAAC	(GAT)7
KNUPF89	ATATCCCATTTTCTGATGCAAG	CCTTTTTCTTGCCTACTCATCA	(AGTG)6
KNUPF107	TAAGGTTGCCGATTGTACTTTT	ACAGAATGGCTCACAAGGATAG	(AG)25
KNUPF112	AGTTGGAGTGGTTAAACTTGGA	CACGCACACTCCAATACTACAA	(AG)17
KNUPF113	GTAGGCATCCCTTTTTCTTCC	TTCAAGTTTACTCTTCACCGCT	(CT)17
KNUPF117	GTTGACCGATTTAGTTGGTTGT	TTTACACTGGTGACCGTCATT	(ATA)7
KNUPF118	CTAAAGATCATTGTGGAGGGAA	ACTCCATGAAATCCAACTCATC	(ATG)7
KNUPF121	ACATACAAATGTCTTTCCTGGG	AAGTAAGACATGGGAATTGGTG	(TCT)7
KNUPF125	TGTCCTTATTTTTCGTCGTCTT	TTCATAGAAACCTATGCCCTTG	(AAAG)4

## Data Availability

Data is contained within the article or Supplementaryary Materials.

## Supplementary Materials

The following are available online at https://www.mdpi.com/article/10.3390/plants10071404/s1, Table S1a: Difference in the contents of five fatty acids between 50 *Perilla* accessions with a high total fatty acid content of 115% or more, Table S1b: Difference in the contents of five fatty acids between 50 *Perilla* accessions with a low total fatty acid content of 100% or less.
