# Supplementary material for: Identifying SSR Markers Related to Seed Fatty Acid Content in Perilla Crop (Perilla frutescens L.)
Source: Plants (Basel). 2021 Jul 9;10(7):1404. doi: 10.3390/plants10071404 (PMC8309404; doi:10.3390/plants10071404)
Supplement: Supplementary file 1 [file plants-10-01404-s001.zip › plants-1277572-supplementary.pdf]

Supplement Table 1a. Difference in the contents of five fatty acids between 50 *Perilla* accessions with a high total fatty acid content of 115% or more.

Accession No.	Fatty Acid Content					Total (%)
	Palmitic Acid (%)	Stearic Acid (%)	Oleic Acid (%)	Linoleic Acid (%)	Linolenic Acid (%)	
104232	7.39	3.47	17.9	15.4	74.4	118.6
104241	7.38	3.19	19.0	15.4	71.7	116.7
104486	7.29	3.36	17.7	16.6	70.5	115.4
104507	7.28	3.13	19.6	20.9	65.9	116.8
104516	8.18	3.26	18.4	20.3	66.4	116.6
104754	8.23	3.28	19.1	22.1	65.9	118.6
105801	8.91	3.33	18.5	21.0	69.7	121.5
105833	7.09	3.41	20.9	15.2	70.5	117.1
105928	8.76	3.24	18.7	18.3	69.8	118.8
105936	7.84	3.20	18.8	15.7	75.0	120.5
105939	7.41	2.56	17.1	20.2	72.2	119.4
108995	7.07	3.14	20.4	19.3	68.1	118.0
109580	7.43	3.34	18.2	17.0	72.4	118.4
110974	7.79	3.30	18.5	14.2	75.1	118.9
113100	7.54	3.21	19.1	20.1	66.3	116.2
113184	8.53	3.29	17.8	21.0	68.6	119.2
113569	7.45	3.06	18.6	20.0	68.8	117.9
117000	7.02	3.77	21.2	13.5	70.8	116.3
117013	7.08	3.47	20.4	15.3	69.5	115.8
117015	7.05	3.88	19.0	10.2	75.4	115.5
117017	6.87	3.78	20.3	19.7	67.3	117.9
117023	7.28	3.70	19.6	12.6	72.9	116.0
117031	7.49	3.95	21.3	12.0	71.0	115.7
117033	6.85	3.97	21.9	15.1	68.8	116.6
117037	7.37	3.79	21.3	14.5	69.1	116.0
117078	6.91	3.62	19.8	13.3	71.7	115.4
117091	7.19	3.28	18.1	20.6	70.3	119.5
117125	7.53	3.71	19.2	16.7	68.0	115.2
117161	8.92	3.36	17.2	16.4	70.5	116.4
117187	7.28	3.25	18.7	13.6	72.5	115.4
121520	6.52	3.72	18.5	13.8	73.8	116.3
157402	7.26	2.95	17.3	20.0	67.9	115.4
157489	7.10	3.99	19.2	12.6	74.4	117.3
157587	9.13	3.29	17.7	23.4	71.3	124.8
158273	7.01	3.01	19.7	21.0	66.0	116.7
158274	8.52	3.12	15.6	20.2	68.7	116.1
175906	7.52	3.06	16.8	17.7	72.5	117.6
180966	7.73	3.10	16.7	16.1	73.8	117.4
180968	6.79	3.69	19.2	17.5	68.6	115.8
180973	7.78	3.14	16.2	17.9	71.1	116.1
180978	8.73	3.12	16.0	19.2	70.9	117.9
181994	7.08	3.18	18.5	17.1	73.1	119.0
185634	8.03	3.24	17.7	21.8	66.3	117.1
210187	7.33	3.34	19.0	20.6	66.6	116.8
210189	7.62	3.35	16.1	14.0	74.7	115.8
213786	8.33	3.33	17.5	16.3	71.7	117.1
213787	7.57	3.01	18.5	12.7	74.8	116.6
214489	7.58	3.13	17.3	18.0	71.1	117.1
214492	8.58	3.28	16.5	17.8	68.9	115.1
214493	7.14	3.67	17.9	13.7	73.3	115.7
Mean	7.60 ± 0.63	3.36 ± 0.30	18.6 ± 1.47	17.2 ± 3.15	70.6 ± 2.77	117.2 ± 1.82
Max	9.13	3.99	21.9	23.4	75.4	124.8
Min	6.52	2.56	15.6	10.2	65.9	115.1

Supplement Table 1b. Difference in the contents of five fatty acids between 50 *Perilla* accessions with a low total fatty acid content of 100% or less.

Accession No.	Fatty Acid Content					Total (%)
	Palmitic Acid (%)	Stearic Acid (%)	Oleic Acid (%)	Linoleic Acid (%)	Linolenic Acid (%)	
104669	7.57	3.10	14.3	14.8	57.9	97.6
110826	7.39	2.87	14.2	15.4	58.1	97.9
117027	7.26	3.37	14.2	15.2	57.9	97.9
117093	7.74	3.53	13.5	15.9	56.5	97.3
117118	7.18	3.06	15.3	15.8	56.9	98.2
117120	6.95	2.91	13.6	14.4	60.3	98.2
117121	7.33	2.96	11.9	14.5	61.0	97.7
117123	7.54	3.27	11.9	17.1	57.7	97.6
117128	7.54	3.24	14.2	15.1	57.7	97.8
117133	7.76	3.38	11.3	15.6	59.1	97.1
117134	7.42	2.67	13.8	16.0	57.7	97.6
117137	7.67	2.95	12.5	17.7	56.6	97.5
117152	7.84	3.22	12.6	16.9	57.1	97.7
117153	7.67	3.28	14.8	17.7	54.3	97.7
117178	7.85	3.33	16.5	12.6	57.6	98.0
117180	7.63	2.86	12.8	15.5	59.3	98.0
117195	7.72	3.02	17.1	14.3	55.8	97.9
117208	7.59	3.03	16.8	14.2	56.1	97.7
157409	7.74	3.14	13.5	14.0	59.4	97.8
157413	7.47	3.21	15.6	15.2	56.3	97.8
157421	7.70	3.02	15.8	15.2	56.4	98.1
157424	7.61	3.22	14.7	17.1	55.2	97.8
157427	7.76	3.16	13.4	13.8	59.5	97.6
157435	7.78	3.18	13.1	14.4	59.2	97.6
157437	7.55	3.09	13.9	14.5	59.0	98.0
157446	7.46	3.11	13.1	16.5	57.4	97.6
157458	7.12	2.70	14.4	14.0	59.9	98.1
157461	7.12	2.67	13.8	14.3	60.3	98.3
157468	7.38	2.71	13.7	14.3	59.7	97.8
157469	6.00	1.90	12.0	21.2	58.8	99.9
157486	6.84	2.95	18.8	13.7	57.0	99.2
157512	7.60	3.17	13.8	15.8	57.3	97.6
157525	7.19	3.11	14.8	14.3	58.5	97.9
157528	7.41	2.93	12.8	14.8	59.8	97.7
157546	7.06	2.64	13.1	14.4	60.4	97.6
157549	6.98	3.19	13.7	12.7	60.7	97.3
157555	7.18	2.82	14.4	13.8	59.3	97.5
157557	7.10	2.84	13.5	13.3	60.7	97.4
157571	7.09	2.71	12.6	15.1	60.6	98.1
157575	7.06	2.49	13.2	13.5	61.8	98.1
157594	6.70	2.59	14.3	12.5	62.3	98.4
157595	6.90	2.55	12.4	14.8	61.5	98.2
158275	7.07	3.14	14.8	15.0	57.7	97.7
177137	5.10	2.10	11.1	22.7	58.9	99.9
180960	6.89	2.81	16.5	14.4	57.2	97.8
185661	7.05	2.95	13.8	14.2	59.9	97.9
191152	7.08	3.03	14.8	14.3	58.2	97.4
209219	7.08	2.93	12.7	14.9	60.3	97.9
209222	6.20	1.70	15.3	17.0	59.7	99.9
214465	7.22	2.87	13.6	14.4	59.9	97.9
Mean	7.26 ± 0.50	2.93 ± 0.35	13.4 ± 1.51	15.2 ± 1.85	58.6 ± 1.78	97.9 ± 0.60
Max	7.85	3.53	18.8	22.7	62.3	99.9

Min	5.10	1.70	11.1	12.5	54.3	97.1
